# Amyloid‐beta 1‐40 is associated with alterations in NG2+ pericyte population ex vivo and in vitro

**DOI:** 10.1111/acel.12728

**Published:** 2018-02-17

**Authors:** Nina Schultz, Kristoffer Brännström, Elin Byman, Simon Moussaud, Henrietta M. Nielsen, The Netherlands Brain Bank, Anders Olofsson, Malin Wennström

**Affiliations:** ^1^ Clinical Memory Research Unit Department of Clinical Sciences Malmö Lund University Malmö Sweden; ^2^ Department of Medical Biochemistry and Biophysics Umeå University Umeå Sweden; ^3^ Department of Neurochemistry Stockholm University Stockholm Sweden; ^4^ Netherlands Institute for Neuroscience Amsterdam The Netherlands

**Keywords:** Alzheimer's disease, amyloid‐beta 1‐40, hippocampus, pericytes

## Abstract

The population of brain pericytes, a cell type important for vessel stability and blood brain barrier function, has recently been shown altered in patients with Alzheimer's disease (AD). The underlying reason for this alteration is not fully understood, but progressive accumulation of the AD characteristic peptide amyloid‐beta (Aβ) has been suggested as a potential culprit. In the current study, we show reduced number of hippocampal NG2+ pericytes and an association between NG2+ pericyte numbers and Aβ1‐40 levels in AD patients. We further demonstrate, using in vitro studies, an aggregation‐dependent impact of Aβ1‐40 on human NG2+ pericytes. Fibril‐EP Aβ1‐40 exposure reduced pericyte viability and proliferation and increased caspase 3/7 activity. Monomer Aβ1‐40 had quite the opposite effect: increased pericyte viability and proliferation and reduced caspase 3/7 activity. Oligomer‐EP Aβ1‐40 had no impact on either of the cellular events. Our findings add to the growing number of studies suggesting a significant impact on pericytes in the brains of AD patients and suggest different aggregation forms of Aβ1‐40 as potential key regulators of the brain pericyte population size.

## 1 INTRODUCTION

One of the most well‐established hallmarks of Alzheimer's disease (AD) is the progressive accumulation of the amyloid‐beta (Aβ) peptide, forming Aβ plaques in the brain. Brain areas containing such Aβ plaques display neuroinflammation, featured by activated glial cells, and substantial loss of neurons. Not only neurons and glial cells are affected in AD, but also the vascular network appears to be altered and reduced vessel density, dysfunctional blood–brain barrier (BBB) and microbleeds are commonly seen in AD patients (Iadecola, 2004; Kalaria, 2002; Zlokovic, 2005). A recent study conducted by Sengillo and colleagues has further showed a marked loss of pericytes in AD patients compared to age‐matched nondemented controls (Sengillo et al., 2013). These cells are very dynamic and versatile. In their mature state, they play a key role in vessel stabilization and vessel permeability, but in response to changes in their environment, pericytes become activated and take part in events such as vascular remodelling, inflammation (Bergers & Song, 2005; Paul et al., 2012) and clearance of neurotoxic substances (including Aβ) (Zlokovic, 2008). Hence, loss of pericytes leads not only to deleterious events, such as impaired BBB or vessel leakage (Quaegebeur, Segura & Carmeliet, 2010), but could also underlie the accumulation of Aβ in the AD brain.

The underlying cause for the loss of pericytes in AD patients is not yet fully understood, but some evidence points towards Aβ. Firstly, the loss of pericytes found in AD patients correlates with increased Aβ depositions (Sengillo et al., 2013). In support, a similar correlation between pericyte number and Aβ load has been found in transgenic APP mouse, an in vivo model of AD (Winkler, Sagare & Zlokovic, 2014). Secondly, in vitro studies show that Aβ influences survival of cultured brain pericytes. However, it is important to point out that Aβ can appear in several different species, whereof Aβ1‐42 and Aβ1‐40 are two of the most abundant forms. Alzheimer′s disease is foremost associated with the former specie, as insoluble Aβ1‐42 fibrils are the major component of Aβ plaques and soluble oligomer Aβ1‐42 is strongly neurotoxic (Dahlgren et al., 2002). The impact of Aβ1‐42 on brain pericytes in the human brain is scarcely investigated, but a few in vitro studies show reduced cell survival after prolonged Aβ1‐42 stimulation (Verbeek, de Waal, Schipper & Van Nostrand, 1997; Wilhelmus et al., 2007) and altered shedding of the pericyte adhesion molecule chondroitin sulphate proteoglycan NG2 in the presence of oligomer and fibril Aβ1‐42 (Schultz, Nielsen, Minthon & Wennstrom, 2014). In comparison with Aβ1‐42, Aβ1‐40 has in several human brain tissue studies been shown to have an impact on pericytes. This Aβ specie is not as prone to aggregate as Aβ1‐42, but can nonetheless form depositions, so‐called cerebral amyloid angiopathy (CAA), in the vessel walls in the brain (Maia, Mackenzie & Feldman, 2007). The CAA is often associated with pericytes displaying degenerative features (Verbeek, Van Nostrand, Otte‐Holler, Wesseling & De Waal, 2000), and thus, it has been hypothesized that aggregated Aβ1‐40 is particularly toxic for pericytes. Studies showing a reduced cell survival of cultured human brain pericytes in presence of Dutch variant of Aβ1‐40 (a mutation promoting aggregation) support this hypothesis (Verbeek et al., 1997).

To conclude, several clinical and preclinical studies show that Aβ influences the pericyte population, but whether either of the Aβ species (i.e. Aβ1‐40 or Aβ1‐42) and whether aggregation forms of the Aβ species play a role in the pericyte loss seen in AD patients remain to be investigated. We therefore analysed the pericyte population in AD patients, in comparison with nondemented controls, by staining postmortem brain tissue against NG2 and laminin alpha (α)5. These two markers are found on different subset of pericytes: NG2 is expressed by activated pericytes on arterioles/capillaries (Stapor, Sweat, Dashti, Betancourt & Murfee, 2014) and laminin is expressed by mature pericytes on arterioles and capillaries as well as postcapillary venules (Yousif, Di Russo & Sorokin, 2013). The association between pericyte numbers and levels of Aβ1‐40 or Aβ1‐42 in brain homogenates was thereafter analysed. We also investigated the direct impact of different aggregations forms of Aβ1‐40 and Aβ1‐42 on proliferation, cell survival and caspase 3/7 activity of cultured human brain pericytes.

## 2 RESULTS

### 2.1 Brain tissue studies

#### 2.1.1 Decreased number of NG2+ pericytes and number of NG2+ pericytes/vessel in AD brains

**Figure 1:**
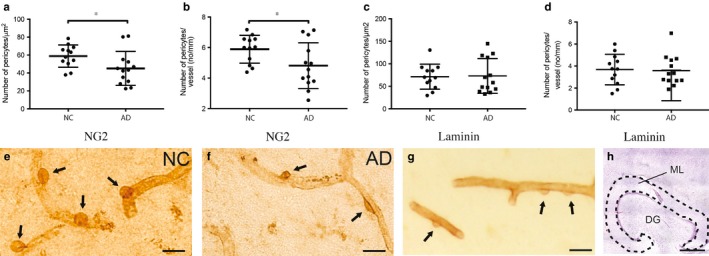
Column scatter plots in (a and c) demonstrate decreased number of NG2+ pericytes/μm^2^ (a) and number of NG2+ pericytes/vessel length (b) in brains from patients with Alzheimer's disease (AD) compared to nondemented controls (NC). Column scatter plots in (c and d) demonstrate unaltered number of laminin+ pericytes/μm^2^ (c) and number of laminin+ pericytes/vessel length (d) in brains from patients with AD compared to NC. Data are analysed using Student's *t*‐test, results are presented as means ± standard deviations. Significant difference at *p < .05. Images in (e and f) show hippocampal pericytes stained against NG2 (indicated by arrows) in nondemented controls (NC) (e) and patients with Alzheimer's disease (AD) (f). Image in (g) shows a representative staining against laminin, where pericytes are indicated by arrows. The area in which pericytes were counted, that is the molecular layer (ML) of hippocampus, is outlined in image (h). Scale bar (e–g) 20 μm. Scale bar (h) 1,000 μm

To examine whether hippocampal pericytes are affected in patients with Alzheimer's disease (AD), we counted the number of NG2+ and laminin+ pericytes and measured the length of vessels in each staining. The molecular layer (ML) hippocampal subarea was selected since capillaries (<10 μm in diameter) in the area are easy to distinguish and amyloid‐beta plaques are present in this area in later stages of AD. We found significantly fewer NG2+ pericytes as well as fewer NG2+ pericytes/vessel length in the ML of AD patients compared to nondemented controls (*F*(1, 20) = 1.506, *p *=* *.039, $\eta_{\rho }^{2}$ = 0.196 and *F*(1, 20) = 5.563, *p *=* *.035, $\eta_{\rho }^{2}$ = 0.204, respectively) (Figure 1a,b), but no alterations in vessel length. However, neither number of laminin+ pericytes nor laminin+ pericytes/vessel length were significantly altered when the two groups were compared (Figure 1c,d). Representative images of NG2+ pericytes and laminin+ pericytes are shown in Figure 1e–g and an image of hippocampus where ML is outlined is shown in Figure 1h. We also investigated whether APOE‐ε4 genotype affects the measured pericyte variables, but we found no differences in either NG2+ or laminin+ pericyte numbers, vessel length or number of pericytes/vessel length when comparing APOE‐ε4 carriers (*n *=* *13) with APOE‐ε4 noncarriers (*n *=* *12). Finally, we found no association between age and any of the measured pericyte variables and no difference in pericyte variables when males were compared with females or when patients with severe vascular disease where compared with patients without severe vascular disease.

#### 2.1.2 Correlations between Aβ1‐40 levels and number of pericytes and number of pericytes/vessel in AD patients

Next, we investigated whether levels of Aβ1‐40 and Aβ1‐42 in the hippocampal samples were associated with the pericyte loss in AD patients. We therefore dissolved PFA‐fixed brain sections in formic acid and measured the total amount of Aβ levels (aggregated + soluble Aβ). We found a trend towards higher levels of Aβ1‐42 and higher Aβ1‐42/40 ratio in the AD patients compared to nondemented controls (17.91 ± 19.59 vs. 4.64 ± 11.91, *p *=* *.061 and 7.39 ± 10.78 vs. 0.78 ± 1.46, *p *=* *.058, respectively). In contrast, levels of Aβ1‐40 were not significantly different when the two groups were compared (10.10 ± 12.31 vs. 5.10 ± 4.98, *p *=* *.209).

**Figure 2:**
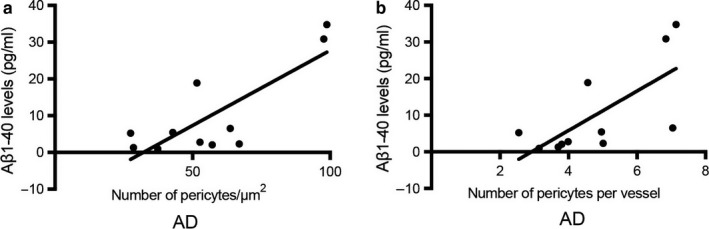
Scatter plots with linear regression lines demonstrating correlations between Aβ1‐40 levels in formic acid‐treated hippocampus sections and number of pericytes/μm^2^ in the molecular layer of hippocampus. Scatter plot in (a) demonstrates the correlation between Aβ1‐40 levels and pericyte numbers/μm^2^ within the AD patient group. Scatter plot in (b) shows the correlation between Aβ1‐40 levels and number of pericytes/vessel length within the AD patient group. Data are analysed using Spearman correlation test

Correlation analysis further showed that levels of Aβ1‐40 and number of NG2+ pericytes significantly correlated when the whole cohort was analysed (*r* = .470, *p *=* *.024). This correlation remained significant when the AD patient group was separately analysed (*r* = .713, *p *=* *.014; Figure 2a), but only a trend to significance was seen when the nondemented control group was analysed (*r* = .504, *p *=* *.095). We also found a positive correlation between Aβ1‐40 levels and the number of NG2+ pericytes/vessel length in AD patients (*r* = .705, *p *=* *.015) (Figure 2b), but a similar association was not found in nondemented controls. Finally, when the whole cohort was analysed, we could see a negative and significant correlation between the Aβ1‐42/40 ratio and the NG2+ number of pericytes (*r* = −0.451, *p *=* *.035) as well as between Aβ1‐42/40 ratio and the number of NG2+ pericytes/vessel length (*r* = −0.544, *p *=* *.009). This correlation was lost when the analysis was performed on the AD and NC groups separately. No significant correlations were found between the brain Aβ1‐42 levels and any of the NG2+ pericyte variables (i.e. the number of pericytes, the length of the vessels and the number of pericytes/vessel), regardless of whether the whole cohort was analysed or the two groups were analysed separately (Figure S1). No correlations between Aβ1‐40, Aβ1‐42 or Aβ1‐42/40 ratio and any of the laminin+ pericyte variables were found, regardless of whether the whole cohort was analysed or the two groups were analysed separately. Furthermore, we found no correlation between the measured NG2+ or laminin+ pericyte variables and ABC amyloid stages or Braak stages for NFT.

### 2.2 Cell culture studies

Since our postmortem studies indicate that Aβ1‐40 levels are associated with changes in NG2+ pericytes numbers in the AD brain, we found it interesting to investigate the direct impact of different aggregation forms of Aβ1‐40 on brain pericyte population. For this purpose, we used NG2‐ and PDGFR‐β‐expressing HBVP as a cell culture model of brain pericytes and analysed alterations in viability, caspase activity and proliferation of these cells after exposure to different aggregation forms of Aβ1‐40 and Aβ1‐42. Analysis of both Aβ preparations used in the experiment showed that the fibril‐enriched preparations (EP) contained higher amount of thioflavin T (ThT)‐incorporated β‐sheets (indicative of fibril formation) compared to oligomer‐EP and monomer preparation. The amount of oligomers (detected with oligomer‐specific antibody ELISA) was higher in the Aβ1‐40 oligomer‐EP compared to monomer and fibril‐EP, whereas the Aβ1‐42 oligomer‐EP and the Aβ1‐42 fibril‐EP contained equally higher amount of oligomers compared monomer preparations (Figure S3). Further TEM analysis of the fibril‐EPs revealed the presence of aggregated straight Aβ1‐40 and Aβ1‐42 fibrils with a length of approximately 100nm and above, whereas the oligomer preparations contained primarily oligomers (Figure S4).

#### 2.2.1 Amyloid‐beta 1‐40 alters viability in an aggregation‐dependent manner

**Figure 3:**
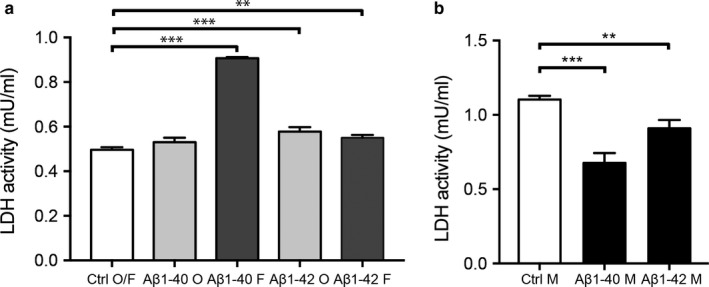
Bar graphs demonstrating alterations in cytotoxicity, measured by LDH assay, in cell culture supernatants from HBVPs after exposure to 10 μm monomer, oligomer‐ or fibril‐EP Aβ1‐40 and Aβ1‐42 for 96 hr. Graph in (a) shows significantly increased LDH activity (i.e. increased cell death) in HBVP exposed to fibril‐EP Aβ1‐40, oligomer‐EP Aβ1‐42 and fibril‐EP Aβ1‐42 compared to Ctrl O/F. Graph in (b) shows the significantly decreased LDH activity (i.e. increased viability) in HBVP exposed to monomer Aβ1‐40 and monomer Aβ1‐42 compared to Ctrl M. Data were analysed using one‐way analysis of variance (ANOVA), followed by Dunnett's post hoc correction (*n *=* *4 comparisons) (oligomer‐ and fibril‐EP Aβ1‐40 and Aβ1‐42) or Student's *t*‐test (monomers Aβ1‐40 and Aβ1‐42). Results are presented as means ± standard deviations. Significant difference at **p <* *.01 and ***p <* *.001

In the viability experiment, we exposed HBVP to monomer and oligomer‐ and fibril‐EP Aβ1‐40 for either 24 or 96 hr. Already at 24 hr, we found significantly increased necrotic cell death (indicated by increased LDH activity) after fibril‐EP Aβ1‐40 exposure compared to Ctrl O/F (0.70 ± 0.03 vs. 0.58 ± 0.05, *p *=* *.003). At this time point, we also found significantly increased viability (indicated by lowered LDH activity) after monomer Aβ1‐40 exposure to Ctrl M (0.55 ± 0.00 vs. 0.62 ± 0.01, *p *=* *.003). Similar results, but with greater differences, were detected after exposure to the three Aβ1‐40 stimuli for 96 hr (Figure 3a,b). Interestingly, no differences in LDH activity were seen after exposure to oligomer‐EP Aβ1‐40 compared to Ctrl O/F, neither after 24‐hr (0.56 ± 0.00 vs. 0.58 ± 0.05, *p *=* *.939) nor after 96‐hr exposure (0.53 ± 0.02 vs. 0.50 ± 0.01, *p *=* *.131) (Figure 3a).

In comparison, no significant differences in LDH activity were detected in cells after 24‐hr exposure of oligomer‐EP Aβ1‐42 and fibril‐EP Aβ1‐42 (0.59 ± 0.04 vs. 0.58 ± 0.05, *p *=* *.994 and 0.61 ± 0.01 vs. 0.58 ± 0.05, *p *=* *.686, respectively). Neither 24‐hr exposure of monomer Aβ1‐42 altered the LDH activity in these cells (0.65 ± 0.23 vs. 0.62 ± 0.01, *p *=* *.109). However, after 96‐hr exposure, significantly increased LDH activity was detected in cells exposed for oligomer‐EP Aβ1‐42 and fibril‐EP Aβ1‐42 (0.58 ± 0.20 vs. 0.50 ± 0.01, *p *=* *.0002; and 0.55 ± 0.01 vs. 0.50 ± 0.01, *p *=* *.005) (Figure 3a). Furthermore, monomer Aβ1‐42 exposure for 96 hr significantly increased the viability (indicated by lowered LDH activity) compared to Ctrl M (0.91 ± 0.06 vs. 1.1 ± 0.03, *p *=* *.008) (Figure 3b).

#### 2.2.2 Amyloid‐beta 1‐40 alters caspase 3/7 activity in an aggregation‐dependent manner

**Figure 4:**
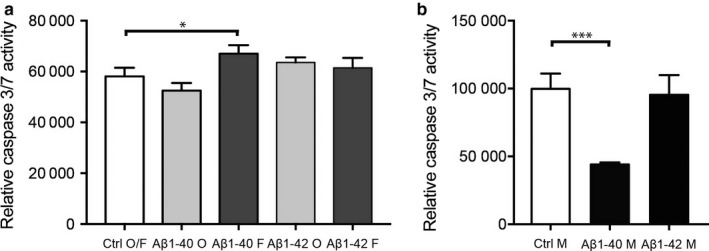
Bar graphs demonstrating alterations in cytotoxicity, measured by caspase 3/7 activity, in HBVPs after exposure to 10 μm monomer, oligomer‐ or fibril‐EP Aβ1‐40 and Aβ1‐42 for 24 hr. Graph in (a) shows the significantly increased relative caspase 3/7 activity in HBVP after exposure of fibril‐EP of Aβ1‐40 compared to Ctrl O/F. Graph in (b) demonstrates the significant increased relative caspase 3/7 activity in HBVP after exposure to monomer Aβ1‐40 compared to Ctrl M. Data were analysed using one‐way analysis of variance (ANOVA), followed by Dunnett's post hoc correction (*n *=* *4 comparisons) (oligomer‐ and fibril‐EP of Aβ1‐40 and Aβ1‐42) or Student's *t*‐test (monomer preparations of Aβ1‐40 and Aβ1‐42). Results are presented as means ± standard deviations. Significant difference at *p <* *.05 and ***p <* *.001

Since previous studies in rat neurons have shown that Aβ25‐35‐induced cell death is mediated via caspases (Harada & Sugimoto, 1999; Marin et al., 2000), we found it interesting to investigate whether exposure to Aβ1‐40 and Aβ1‐42 also affects caspase activation. We therefore analysed activity of caspases 3 and 7 (the two most prominent executor caspases in the apoptosis pathway (Elmore, 2007)) in HBVP after exposure to the three different aggregation forms of Aβ1‐40 and Aβ1‐42. Our analysis showed significantly increased caspase 3/7 activity after 24‐hr exposure to fibril‐EP Aβ1‐40 compared to Ctrl O/F (67032.83 ± 3342.54 vs. 58101.83 ± 3395.34, *p *=* *.021; Figure 4a), but unaltered caspase 3/7 activity after 24‐hr exposure of oligomer‐EP Aβ1‐40 compared to Ctrl O/F (52530.17 ± 2988.63 vs. 58101.83 ± 3395.34, *p *=* *.170; Figure 4a). The caspase 3/7 activity assay did not detect any significant alterations after 24‐hr exposure of either oligomer‐EP Aβ1‐42 compared to Ctrl O/F (63597.33 ± 1995.12 vs. 58101.83 ± 3395.34, *p *=* *.177) or fibril‐EP Aβ1‐42 compared to Ctrl O/F (61416.00 ± 3981.95 vs. 58101.83 ± 3395.34, *p *=* *.550; Figure 4a). Exposure of monomer Aβ1‐40 instead led to significantly decreased caspase 3/7 activity compared to Ctrl M (44099.83 ± 1444.16 vs. 99770.50 ± 11318.10, *p *=* *.001; Figure 4b). No significant difference in caspase 3/7 activity was detected after 24‐hr exposure of monomer Aβ1‐42 compared to Ctrl M (95384.00 ± 14614.34 vs. 99770.50 ± 11318.10, *p *=* *.839; Figure 4b).

#### 2.2.3 Aβ1‐40 alters proliferation in an aggregation‐dependent manner

**Figure 5:**
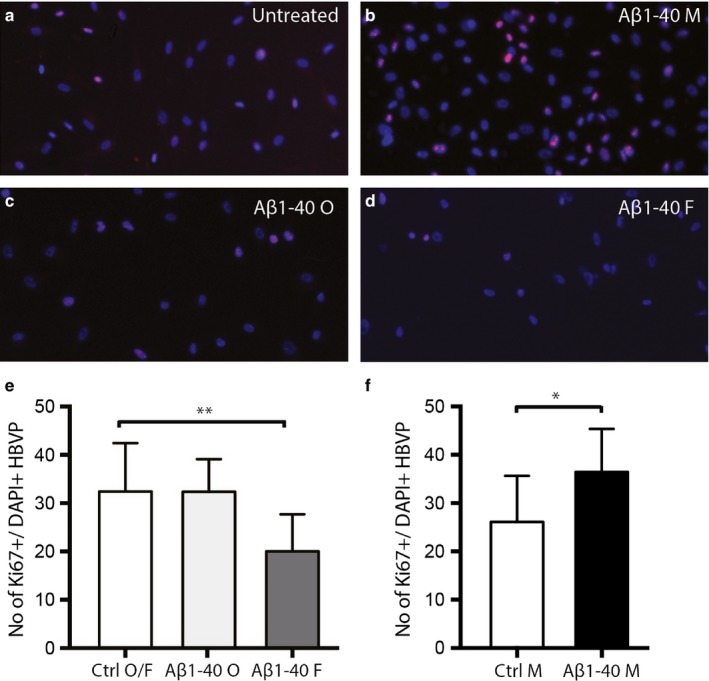
Bar graphs demonstrating alterations in Ki67+ cells, indicative of cell proliferation, in HBVPs after exposure to 10 μm monomer, oligomer‐ or fibril‐EP Aβ1‐40 for 24 hr. Image in (a) shows untreated HBVP stained against Ki67 and DAPI. Image in (b) demonstrates Aβ1‐40 monomer‐exposed HBVP stained against Ki67 and DAPI. Image in (c) demonstrates HBVP stained against Ki67 and DAPI after exposure to oligomer‐EP Aβ1‐40. Image in (d) shows fibril‐EP Aβ1‐40‐exposed HBVP stained against Ki67 and DAPI. Graph in e) shows the significantly decreased number of Ki67+ HBVPs after exposure of fibril‐EP of Aβ1‐40 compared to Ctrl O/F. Graph in (f) demonstrates the significantly increased number of Ki67+ HBVPs after exposure of monomer EP of Aβ1‐40 compared to Ctrl M. Data were analysed using one‐way analysis of variance (ANOVA), followed by Dunnett's post hoc correction (*n *=* *2 comparisons) (oligomer‐ and fibril‐EP of Aβ1‐40) or Student *t*‐test (monomer preparations of Aβ1‐40). Results are presented as means ± standard deviations. Significant difference at *p <* *.05, **p <* *.01

Next, we investigate the impact of the three different aggregation forms of Aβ1‐40 and Aβ1‐42 on proliferation of HBVPs by the use of staining against the endogenous proliferation marker Ki67. Representative images showing the Ki67 staining of untreated HBVP and HBVP after 24‐hr exposure to monomer, oligomer‐EP and fibril‐EP of Aβ1‐40 are shown in Figure 5a–d. Analysis of this staining showed that the number of Ki67+ HBVPs significantly decreased after 24‐hr exposure to fibril‐EP Aβ1‐40 compared to Ctrl O/F (0.20 ± 0.07 vs. 0.32 ± 0.10, *p *=* *.005; Figure 5e), but no detectable changes in number of Ki67+/DAPI+ HBVP were detected after exposure to oligomer‐EP Aβ1‐40 (0.32 ± 0.07 vs. 0.32 ± 0.10, *p *=* *.992; Figure 5e). Exposure to the monomer Aβ1‐40 on the other hand induced a significant increase in numbers of Ki67+ HBVPs compared to Ctrl M (0.36 ± 0.09 vs. 0.26 ± 0.01, *p *=* *.022; Figure 5f). No significant differences in number of Ki67+/DAPI+ HBVP were found after 24‐hr exposure to Aβ1‐42 monomer, Aβ1‐42 oligomer‐EP or Aβ1‐42 fibril‐EP compared to their respective control (0.19 ± 0.04 vs. 0.18 ± 0.05, *p *=* *.795; 0.16 ± 0.01 vs. 0.17 ± 0.01, *p *=* *.620 and 0.18 ± 0.05 vs. 0.17 ± 0.01, *p *=* *.867, respectively).

## 3 DISCUSSION

Studies analysing brain pericytes by the use of immunohistochemical staining are upon recently very few and foremost performed on rodents. The reason for this shortage of studies are difficulties in staining pericytes in human brain tissue. Many of the pericyte‐specific markers are sensitive for overfixation and antigen retrieval methods used in stainings of paraffin sections. Our analysis of number of pericyte cell bodies in the ML of hippocampus was performed by the use of immunohistochemical stainings against the pericyte marker chondroitin proteoglycan NG2 and the pericyte/endothelial marker laminin α5. Both markers are expressed by pericytes on arterioles and capillaries (Stapor et al., 2014), but laminin α5 is additionally expressed by pericytes on postcapillary venules (Yousif et al., 2013). Importantly, pericytes are dynamic and versatile and the expression of markers, which includes also nestin, desmin, CD13, PDGFR‐ß and RGS5, fluctuates depending on activation, migration and vessel‐stabilization pericyte state. For example, NG2 is associated with both mature and active pericytes (migration, vascular remodelling and inflammation; Stapor et al., 2014), whereas pericytes expressing laminin are foremost found on quiescent and mature vessels (Hallmann et al., 2005). None of the markers mentioned above are found on all pericytes, and hence, immunohistochemistry based on one marker only enables analysis of a subset of the pericyte population. Our study showed significantly decreased number of NG2+ pericytes as well as decreased number of NG2+ pericytes/vessel length in AD patients compared to nondemented controls, but the analysis of laminin+ pericytes showed no significant differences in pericyte variables between the groups. This difference highlights the heterogeneity of the pericyte population (i.e. marker expression and localization) and proposes an impact of AD pathology on foremost activated pericytes. The reduction in NG2+ pericytes could be due to a direct impact on the pericyte population either by degeneration or formation of new NG2+ pericytes, but in view of our laminin result we cannot exclude the possibility that the pericyte population size remains the same and the difference is due to a downregulation of NG2 expression. Nevertheless, the results found in our study are in line with the previous study by Sengillo and colleagues, demonstrating a pericyte loss in AD patients (Sengillo et al., 2013). In that study, the number of mural cells (an umbrella term for smooth muscle cells and pericytes) was analysed by measuring the amount of PDGFR‐β+ (a marker for migrating immature mural cells (Song, Ewald, Stallcup, Werb & Bergers, 2005)) and CD13+ (marker for activated pericytes (Svensson, Ozen, Genove, Paul & Bengzon, 2015)) cells associated with vessels/capillaries in hippocampus. They found a 60% reduction in hippocampal mural cells and a 33% reduction in hippocampal pericytes, which is in the order of magnitude with our own study (approximately 26% reduction in NG2+ pericytes). In view of these findings, we conclude that different subsets of pericytes involved in migration, activation and remodelling (NG2+, PDGFR‐β+ and CD13+ pericytes) are affected by AD pathology, whereas mature and quiescent pericytes (laminin+) are less affected.

As mentioned in the introduction, previous studies have suggested Aβ, in particular Aβ1‐40, to be toxic for pericytes (Verbeek et al., 1997; Wisniewski, Wegiel, Wang & Lach, 1992). We therefore sought to investigate whether Aβ1‐40 and Aβ1‐42 levels are associated with the reduced number of pericytes in AD. For this purpose, we analysed levels of Aβ1‐40 and Aβ1‐42 in PFA‐fixed brain sections treated with formic acid. This method both liberates Aβ and dissolves Aβ aggregates. Hence, the samples contain the total amount of Aβ and it is not possible to determine the ratio of monomeric vs. aggregated Aβ. As expected, analysis of the samples showed higher levels of Aβ1‐42 and higher Aβ1‐42/40 ratio in AD patients compared to nondemented controls. Also, higher levels of Aβ1‐40 were noted in AD patients, but this increase was not significant compared to NC. Further, when all individuals as well as only the AD patient group were analysed, a positive and significant correlation between the Aβ1‐40 levels and the number of NG2+ pericytes was found. This finding suggests that Aβ1‐40 has a beneficial role on the NG2+ pericyte population size. If this holds true, it is important to not ignore the fact that the number of NG2+ pericytes decreased in a group (AD) also displaying increased, albeit not significant, Aβ1‐40 levels. A possible explanation to this contradictive result might be found when considering the ratio of monomer vs. aggregated Aβ1‐40 in the samples before FA treatment. Since aggregated Aβ1‐40 has been shown to be toxic (Maia et al., 2007) and monomer is potentially beneficial (as described in our in vitro studies), we speculate that samples from AD patients with lower number of NG2+ pericytes also contain higher portions of toxic aggregated Aβ1‐40 and less of the beneficial monomer Aβ1‐40. This would be in line with previous studies on CSF demonstrating a relationship between lower levels of Aβ1‐40 (indicating increased trapped aggregated Aβ1‐40 in the brain) and increased white matter changes (indicating vascular damage; Janelidze et al., 2016; van Westen et al., 2016).

Negative correlations were also found between Aβ1‐42/40 ratio and number of pericytes as well as with the number of pericytes/vessel length when the whole cohort was analysed. Since the ratio between Aβ1‐42/40 reflects the drive towards increased Aβ1‐42 production at the expense of Aβ1‐40 production, such results could imply that increased Aβ1‐42 levels in relation to Aβ1‐40 proportions underlie the decreased number of NG2+ pericytes in AD patients. In vitro studies (including our current own study) demonstrating increased cell death of cultured pericytes in response to Aβ1‐42 are in line with this hypothesis. However, as the correlations between Aβ1‐42/40 ratio and the measured pericyte variables were not detected when the AD and NC groups were analysed separately, we suspect that the negative correlations in the whole cohort are foremost due to the fact that the measured pericyte variables decrease and the Aβ1‐42/40 ratio increases in AD patients. Moreover, no correlations between Aβ1‐42 and the measured pericyte variables were detected. Furthermore, CSF studies suggest that there is no relationship between Aβ1‐42 and white matter changes (van Westen et al., 2016). We therefore conclude that our findings point towards a specific effect of Aβ1‐40 on the pericyte population. Importantly, it should be noted that there are limitations with our brain tissue study. The cohort size is rather small and therefore studies on larger cohorts are needed in order to confirm our results. Moreover, despite the fact that the individuals included in the NC group were diagnosed as nondemented, some of them displayed Aβ plaque and tau pathology. This may have affected the found results and we cannot rule out the possibility that even greater differences between NC and AD patients could be found if the study was performed on NC individuals completely lacking AD pathology. Our hypothesis that lower number of NG2+ pericytes in AD is due to higher portions of toxic aggregated Aβ1‐40 and less of the beneficial monomer Aβ1‐40 is highly speculative and needs to be verified. However, pericyte numbers in ex vivo studies represent only a snapshot of the summation of the impact of different events (e.g. cell death and proliferation) on pericytes at time of death. This, in combination with the difficulties in identifying the aggregation composition in our brain samples, urged us to instead investigate the direct impact of the different aggregations forms of Aβ1‐40 on cell death and proliferation by the use of cell culture studies. The result of this cell culture study showed that the fibril form of Aβ1‐40 induces cell death (necrosis measured by LDH), 3/7 caspase activity (apoptosis) and reduced proliferation of HBVP. This finding goes well with the previously mentioned study demonstrating degenerated pericytes adjacent to fibril Aβ1‐40‐formed CAA (Maia et al., 2007). The finding is also in line with several cell cultures studies demonstrating that the fibril form of Aβ1‐40 is toxic to neurons (Dahlgren et al., 2002; Okada, Ikeda, Wakabayashi, Ogawa & Matsuzaki, 2008; Walsh, Hartley, Condron, Selkoe & Teplow, 2001), endothelial cells (Hernandez‐Guillamon et al., 2010; Qosa, LeVine, Keller & Kaddoumi, 2014) and smooth muscle cells (Fossati et al., 2010). However, fibril preparations most likely also contain small portion of protofibrils (the first elongated unit appearing in fibril formation; Walsh, Lomakin, Benedek, Condron & Teplow, 1997), and since protofibrils of Aβ have some neurotoxic features (although the Aβ1‐42 protofibrils are substantially less toxic compared to Aβ1‐42 oligomers (Ahmed et al., 2010)), it is important to reflect upon the possibility that also our fibril preparations contain toxic protofibrils. The definition of a protofibril is difficult as it is an intermediate form arising from a transient change in structure. Several researchers have defined protofibrils based on length, where some (Dubnovitsky et al., 2013) regard fibrils below 220 nm as protofibrils, whereas others (Gouwens, Makoni, Rogers & Nichols, 2016; Toyama & Weissman, 2011) have set the‐cut off at 100 nm. Also, fibril morphology and distribution have been used to distinguish the different forms, where individually dispersed fibrils with a curvilinear feature have been defined as protofibrils (Hartley et al., 1999; O'Nuallain et al., 2010; Paranjape, Gouwens, Osborn & Nichols, 2012; Relini et al., 2010). Our fibril preparations contained straight, aggregated fibrils, which were approximately 100 nm and above. In addition, our OMAB and ThT analysis demonstrated lower amount of oligomer and higher amount of β‐sheets in fibril‐EP compared to oligomer‐EP. We therefore draw the conclusion that our fibril‐EP contains high amounts of fibrils, but due to the length of our fibrils, we cannot exclude the possibility that a portion of those fibrils are protofibrils.

Our results further showed that the HBVP viability, 3/7 activity and proliferation were not affected by the oligomer form of Aβ1‐40. This is particularly interesting since the oligomer form of Aβ1‐42, and not the fibril form of this specie, is regarded as the most toxic form (Benilova, Karran & De Strooper, 2012). Our own comparative study showed that stimulation with both oligomer‐EP Aβ1‐42 and fibril‐EP Aβ1‐42 decreased HBVP viability measured by LDH. This result should be viewed from the fact that Aβ1‐42 is more prone than Aβ1‐40 to form alternative structures in the path towards a fibril. This feature results in more heterogeneous preparations and analysis of our preparations revealed the presence of oligomers in both oligomer‐EP and fibril‐EP Aβ1‐42 preparations, whereas fibrils were only found in the fibril‐EP Aβ1‐42 preparation. We are therefore unable to unambiguously show the impact of Aβ1‐42 fibrils on HBVP viability, but the increased cell death seen in response to oligomer‐EP Aβ1‐42 exposure is in line with previous studies (Benilova et al., 2012), highlighting the different impact of the two Aβ species in regard to oligomer toxicity.

In contrast to fibril‐EP Aβ1‐40, our in vitro results showed that monomer Aβ1‐40 both had a rescuing and mitogenic impact on HBVPs. Similar beneficial effect of Aβ1‐40 has been described before. For example, Aβ1‐40 prevents degradation of cell membrane lipids by inhibiting auto‐oxidation of CSF and plasma lipoproteins (Kontush et al., 2001), an event known to be increased in AD patients (Bassett et al., 1999). The Aβ1‐40 peptide is further the most effective Aβ specie to rescue cultured neurons which are dying in response to endogenous Aβ inhibition (Plant, Boyle, Smith, Peers & Pearson, 2003). Our own cell viability results, showing decreased cell death as well as decreased caspase 3/7 activity after monomer Aβ1‐40 exposure, support the idea that Aβ1‐40 is beneficial for cell survival. But our results also show that it is foremost the monomers that have this ability. Apparently, the Aβ1‐40 monomers also have a strong mitogenic impact on HBVPs as our proliferation assay showed marked increased cell division in response to the stimulus. This finding is backed up by a previous study demonstrating a mitogenic property of monomer Aβ1‐40 on cultured human skin fibroblasts (Theda, Drews, Zitnik, Oshima & Martin, 2016), a cell type sharing both origin and certain markers with the pericyte (Birbrair et al., 2013). Interestingly, decreased caspase activity has been associated with hyperproliferation of pancreatic β‐cells in mice (Woo et al., 2003). It may thus be that the decreased activity of caspase 3/7 in response to monomer Aβ1‐40 seen in our study contributes to both the increased cell viability and the increased cell division. Finally, it is important to point out that cell cultures can never replicate a biological system and although pericyte proliferation does occur in the human brain (Fernandez‐Klett et al., 2013; Goritz et al., 2011; Matsushita et al., 2015; Paul et al., 2012), the conditions and rate in the brain are not comparable to the same in a Petri dish. Hence, we would like to stress that our cell model is useful foremost as a tool to demonstrate that differences in pericyte response to Aβ depend on both aggregation form and specie.

## 4 CONCLUSIONS

To conclude, our results confirm the previous study demonstrating pericyte alterations in AD patients and highlight Aβ1‐40 as a potential regulator of brain pericyte population. Moreover, our in vitro studies show that the regulatory role of Aβ1‐40 is dependent on aggregation form, where the monomeric appears to have a rescuing and mitogenic impact, oligomers are quiescent and fibrils induce toxicity. This is particularly interesting given the common notion that the oligomer form of the kin peptide, Aβ1‐42, is considered to be the culprit behind neuronal loss in AD. Hence, our results point out the differences between Aβ species and their aggregation forms in terms of toxicity on pericytes, a finding important to take into consideration when targeting the aggregation process of Aβ as a treatment for AD.

## 5 EXPERIMENTAL PROCEDURES

### 5.1 Individuals included in the study

The study includes frozen samples of midlevel hippocampus from clinically and postmortem‐verified patients with AD (*n *=* *13) and nondemented controls (*n *=* *9) (Netherlands Brain Bank (NBB)). Neuropathological assessments were performed by NBB according to Braak stages of neurofibrillary tangles (NFT) spreading (Braak & Braak, 1995) and Lewy bodies (LB) (Braak et al., 2003) and ABC staging for amyloid (Aβ) (Schultz, Hubbard, Rub, Braak & Braak, 2000). Individuals which after clinical evaluation were considered nondemented and after neuropathological NFT evaluation were scored as 3 or less were considered as nondemented controls. None of the patients included in the study displayed CAA or LB pathology in hippocampus. Individuals with hypertension in combination with either of the following criteria (i) transient ischaemic attack/stroke, (ii) valvular heart disease, (iii) ischaemic heart disease or (iv) arrhythmias were considered as severe cardiovascular disease (CVD) patients. Single nucleotide polymorphisms at positions rs429358 and rs7412 of the *APOE* gene were determined by polymerase chain reactions using allele‐specific primers. Demographic data and the neuropathological assessment of the individuals are presented in Table 1. Written informed consent for the use of brain tissue and clinical data for research purposes was obtained from all patients or their next of kin in accordance with the International Declaration of Helsinki. Medisch Ethische Toetsingscommissie (METc) of VU University has approved the procedures of brain tissue collection, and the regional ethical review board in Lund has approved the study.

**Table 1:** Demographic data and the neuropathological assessment of the individuals included in the study

Clinical diagnosis^a^	Gender	Age (years)	Neuropat. Ev (NFT/Aβ/LB)^b^	APOE genotype	Cause of death	Severe CVD
NC	F	75	1/A/0	2/3	Euthanasia	+
NC	F	60	0/O/0	3/2	Metastasized breast cancer	−
NC	M	55	0/O/0	3/3	Euthanasia with oesophageal cancer	−
NC	M	75	1/A/0	3/3	Cardiac arrest with COPD	+
NC	F	84	2/B/0	3/3	Pulmonary dysfunction	−
NC	F	72	1/A/0	3/3	Euthanasia, met. ovarian cancer	−
NC	F	75	1/0/0	3/3	Euthanasia	−
NC	M	81	3/C/0	4/3	Pancreas carcinoma	−
NC	M	102	3/A/0	4/3	Ileus	+
NC	F	92	3/0/1	4/3	Heart failure	+
NC	M	70	1/0/3	3/3	Pneumonia	+
NC	F	68	0/0/0	3/3	Euthanasia	−
AD	F	91	4/C/0	3/3	Cerebrovascular accident	−
AD	M	85	4/C/0	3/3	Cardiac arrest	+
AD	F	96	4/B/0	3/3	Heart failure, dementia	+
AD	F	87	4/C/0	3/3	Pneumonia	+
AD	M	83	3/B/0	4/3	Dehydration, AD	+
AD	F	88	5/C/0	3/4	Pneumonia	−
AD	F	78	6/C/0	3/4	Cachexia, Dementia	+
AD	M	82	5/C/0	3/4	AD with delirium	+
AD	M	69	6/C/0	4/3	Pneumonia	−
AD	F	92	6/C/0	4/3	Atrioventricular block, severe AD	+
AD	F	70	6/C/0	4/4	Cachexia by dementia	+
AD	F	64	4/C/0	4/4	Euthanasia, AD	−
AD	F	71	5/C/0	4/3	Dehydration, cachexia, dementia	+

F, female; M, male; Severe CVD, cardiovascular disease.

a Individuals clinically diagnosed with Alzheimer's disease (AD) and nondemented controls (NC) included in the study.

b Braak staging of neurofibrillary tangles (NFT) and Lewy bodies (LB) and ABC staging of amyloid (Aβ).

### 5.2 Immunostaining procedures of brain tissue

The frozen hippocampal samples were fixed in 4% paraformaldehyde (PFA) for 4 hr and left in phosphate‐buffered saline (PBS) containing 30% sucrose until they were sunken. The tissue was then cut into 40‐μm‐thick sections using a Microtome Leica and stored free floating in antifreeze cryoprotectant solution at −20°C until analysis. In order to examine the number of pericytes, the brain sections were immunohistochemically (IHC) stained against either the pericyte marker NG2 (which clearly reveals pericyte cell bodies and the whole length of vessels) or laminin α5 (which is expressed by both pericytes and endothelial cells (Yousif et al., 2013)). The sections were quenched in 3% H_2_O_2_ and 10% methanol for 30 min and incubated in Impress reagent kit blocking solution (Vector Laboratories, Burlingame, RI) for 1 hr at room temperature (RT), followed by incubation with mouse‐anti‐NG2 (MAB2029; Millipore, Darmstadt, Germany) or laminin (clone 4C7, Dako, Glostrup, Denmark) in blocking solution overnight at 4°C. Sections were then incubated with Ig Impress reagent kit secondary anti‐mouse antibody (Vector Laboratories) for 2 hr at RT followed by peroxidase detection for 2 min (0.25 mg/ml diaminobenzidine and 0.012% H_2_O_2_). The number of NG2+ and laminin+ pericytes and the length of the vessels were analysed in pictures taken by an Olympus AX70 light microscope equipped with 20 X objectives. Two pictures (345 × 440 mm) within the molecular layer (ML) from three sections of each individual (6 fields in total) were captured. The number of visible NG2+ and laminin+ pericyte cell bodies in the pictures was manually counted by a blinded observer, and the vessel lengths were measured using CellSens Dimension software length measurement tool. The number of pericytes/vessel length was thereafter calculated. Values were averaged and presented as mean number of pericytes/μm^2^, mean length of vessels/μm^2^ and mean number of pericytes/vessel length. To confirm that the NG2 staining reveals the whole length of vessels, we double immunofluorescence stained against NG2 together with the endothelial markers ICAM‐1 and CD34. Sections from AD and NC were blocked in 5% goat serum for 1 hr at RT and before incubating overnight at 4°C with primary antibodies (mouse‐anti‐NG2 and rabbit‐anti‐ICAM‐1 [R&D Systems, Minneapolis, MN] or mouse‐anti‐NG2 and rabbit‐anti‐CD34 [Abcam, Cambridge, UK]). The sections were then incubated with secondary antibodies goat‐anti‐mouse Alexa488 and goat‐anti‐rabbit Alexa594 (Life Technologies, Carlsbad, CA) for 2 hr at RT and thereafter mounted with Vectashield Set mounting medium containing DAPI (Vector Laboratories, Burlingame, CA). The staining showed complete overlap between NG2 and the two markers in terms of vessel length (Figure S2).

### 5.3 Analysis of Aβ l‐40 and Aβ l‐42 in brain tissue

In order to measure the Aβ levels, three brain sections from each individual (*n *=* *12 Ctrl and *n *=* *11 AD) were placed in 100% formic acid (FA) (10 μl/mg brain tissue) overnight (ON). This treatment dissolves aggregated Aβ and hence the FA‐treated samples contain total amount of Aβ levels. The Aβ‐FA solution was then centrifuged at 13,000 × *g* for 20 min at 4°C before the supernatant was lyophilized and redissolved in DMSO. Thereafter, levels of Aβ1‐38, Aβ1‐40 and Aβ1‐42 were analysed using MesoScale Discovery V‐plex Aβ Peptide Panel 1 kit with electrochemiluminescence detection technology (MesoScale Discovery, Rockville, MD) according to manufacturers' protocol. The majority of the Aβ_1‐38_ values were below detection and will therefore not be reported in this study. The electrochemiluminescence signal was quantified using a MesoScale Discovery SECTOR Imager 6000.

### 5.4 Cell culture studies

#### 5.4.1 Cells

Primary foetal human brain vascular pericytes (HBVPs) (ScienCell Research Laboratories) were grown in pericyte cell culture medium (PM) (ScienCell Research Laboratories) containing 2% foetal bovine serum. The cells were grown as monolayers in poly‐L Lysine (PLL)‐coated culture flasks in humidified air with 5% CO_2_ at 37°C until 80%–90% confluent. The morphology of the cells corresponded well to previously published images of HBVP (Bouchard, Shatos & Tracy, 1997; Verbeek, Otte‐Holler, Wesseling, Ruiter & de Waal, 1994), and approximately 99% of the total number of DAPI+ cells (*n *=* *300) were also positive for NG2 and PDGFβR. Prior to experiment, cells were seeded at a density of 20,000 cells/ml using 150 μl of medium per well in Cytostar T‐assay Scintillating 96 wells (Perkin Elmer), flat clear bottom white 96 wells (Sigma‐Aldrich) or 8‐well chamber slides (Lab Tek) and grown until 50% confluent. The medium was changed every 2 days.

### 5.5 Aβ1‐40 and Aβ1‐42 preparations

The Aβ1‐40 peptide (AlexoTech AB, Umeå, Sweden) was dissolved in cold hexafluoro‐2‐propanol (HFIP) (Sigma‐Aldrich, St. Louis, MO), aliquoted, speed‐vacuum dried and stored at −80°C until use. Prior to cell experiments, fibril‐ and oligomer‐enriched preparations (EP) of Aβ1‐40 and Aβ1‐42 were generated according to previously published protocol (Brannstrom et al., 2014). The Aβ1‐40 and the Aβ1‐42 peptides were solubilized in 10 mm NaOH pH 11, and then the pH was adjusted to pH 7.0 by further diluting the solution to a concentration of 100 μm in phosphate buffer. Fibril‐EP was generated by a 72‐hr incubation with agitation in 37°C and oligomer‐EP was generated by 20‐min incubation with agitation RT. Monomer preparations of Aβ1‐40 and Aβ1‐42 were generated by dissolving the peptide in DMSO to the concentration of 2.5 mm and then further diluted to a concentration of 100 μm in phosphate buffer. Prior to cell experiments, fibril, oligomer and monomer preparations were generated by diluting to the 100 μm preparations to the working concentration of 10 μm in pericyte medium. The presence of high amounts of oligomers in the oligomer‐EP Aβ1‐40 and Aβ1‐42 was confirmed using an indirect ELISA with oligomer monoclonal antibody (OMAB) according to previously published protocol (Lindhagen‐Persson, Brannstrom, Vestling, Steinitz & Olofsson, 2010; Figure S3a,b). A thioflavin T (ThT) assay was used to confirm the presence of high amounts of β‐sheets in the fibril‐EP Aβ1‐40 and Aβ1‐42 preparations (Figure S3c,d). The ThT was conducted by incubating 5 μm Aβ1‐40 and Aβ1‐42 preparations together with 40 μm ThT (Millipore, Darmstadt, Germany) in 20 mm phosphate buffer pH 7.4, 150 mm NaCl. The emission at 435 nm and an excitation at 480 nm were measured using the Microplate Spectrophotometer Infinite M200 and the Magellan version 3.5 software. Transmission electron microscopy (TEM) analysis was also performed to verify the presence of oligomers and fibrils in the Aβ1‐40 and Aβ1‐42 preparations (Figure S4a–d). A total of 3.5 μl of the different preparations of Aβ1‐40 and Aβ1‐42 was absorbed for 2 min onto glow‐discharged carbon‐coated copper grids. The samples were washed with water and immediately stained in 50 μl of 1.5% uranyl acetate solution for 30 s. Negatively stained samples were examined using a JEM1230 transmission electron microscope (JEOL) at 80 kV. Transmission electron micrographs were recorded using Gatan UltraScan 1,000 2k × 2k pixel CCD camera and digitalmicrograph ^™^ software (Gatan, Pleasanton, CA).

### 5.6 Cell culture treatment

The medium was removed 2 hr prior to experiments and replaced with serum‐free medium in order to synchronize the cells. After serum starvation, the medium was once again removed and replaced with either serum‐free medium (for the toxicity experiments) or serum‐supplemented medium (for the proliferation experiments) containing either 10 μm fibril‐EP Aβ1‐40, 10 μm oligomer‐EP Aβ1‐40, 10 μm monomer Aβ1‐40, 10 μm fibril‐EP Aβ1‐42, 10 μm oligomer‐EP Aβ1‐42, 10 μm monomer Aβ1‐42 or their respective controls, that is vehicle used for oligomer‐ and fibril‐EP Aβ1‐40 and Aβ1‐42 (NaOH/PBS) or vehicle used for monomer Aβ1‐40 and Aβ1‐42 (DMSO/PBS). These controls will be referred to as Ctrl O/F and Ctrl M, respectively. All experiments were performed in duplicate and repeated independently three times. The cells were incubated in 37°C for 24 or 96 hr depending on the assay. Cell culture supernatant was collected after treatment, centrifuged (275 × *g* 5 min, 4°C), aliquoted and stored at −80°C until used. Cells were lysed using cell lysis kit (Sigma‐Aldrich), aliquoted and stored at −80°C until used.

### 5.7 Analysis of cytotoxicity

To evaluate cytotoxic effect, we used two different cytotoxicity assays (i) extracellular lactate dehydrogenase activity (LDH) (measures foremost necrosis) and (ii) caspase 3/7 activity (measures foremost apoptosis). The levels of LDH activity were measured in conditioned medium using LDH assay kit (Sigma‐Aldrich). In 96‐well plates (Nunc), 100 μl HBVPs cell‐free supernatants were mixed with 100 μl reagent buffer, agitated 30 s 300–450 rpm and then incubated 30 min at RT. The absorbance was measured at 490 nm using Microplate Spectrophotometer Infinite M200 and the Magellan version 3.5 software. Caspase 3/7 activity was analysed using Caspase‐Glo 3/7 luminescence assay according to the manufacturer's instructions (Promega, Fitchburg, WI).

### 5.8 Proliferation analysis

Proliferation of HBVP was analysed by staining HBVPs against the proliferation marker Ki67 after 24‐hr exposure of fibril‐EP, oligomer‐EP or monomers Aβ1‐40 and Aβ1‐42. Cells were fixated with 2% formaldehyde for 15 min, blocked 30 min at RT with 1% BSA, 2.5% Triton‐X, 5% goat serum in PBS. Rinsed twice before incubating with primary antibody (rabbit‐anti‐Ki67, 1:200, Abcam) 60 min at RT. Cells were incubated 1 hr at RT in dark with secondary antibody (1:500, rabbit‐anti‐goat Dylight 459). Vectashield Set mounting medium with DAPI (Vector Laboratories) was used to mount the cells. Proliferation was analysed by counting the number of Ki67+ cells of total DAPI+ cells.

### 5.9 Statistical analysis

Statistical analysis was performed using SPSS software (version 24 for Mac, SPSS Inc., Chicago, IL). The Kolmogorov–Smirnov test was used to assess normal distribution. Since number of pericytes, vessel length and the ratio of pericytes/vessel length were normally distributed, differences between diagnose groups were analysed by the use of independent‐samples *t*‐test. Levels of Aβ1‐40 and Aβ1‐42 as well as Aβ1‐42/40 ratio were not normally distributed, and thus, Mann–Whitney U‐test was used when comparison analysis of these values was performed. Consequently, correlations between the investigated variables in the brain study were examined using the Spearman correlation test. Differences between Ctrl O/F and oligomer‐ and fibril‐EP in the in vitro study were analysed using one‐way analysis of variance (ANOVA), followed by Dunnett's post hoc correction (comparisons for *n *=* *4), whereas the comparisons between monomer preparations of Aβ1‐40 and Aβ1‐42 and their control were analysed using Student's *t*‐test. Results are presented as means ± standard deviations, and a value of *p *<* *.05 level was considered statistically significant.

## AUTHORS' CONTRIBUTION

NS carried out the experiments, analysed data and drafted the manuscript; EB participated in scientific discussions and tissue preparation; KB and AO were involved in preparing and evaluating Aβ peptides; SM and HMN performed *APOE* genotyping; NBB supplied the brain tissue, neuropathological analyses, discussions regarding tissue pretreatment, interpreting and matching clinical data and advising on the approach of the study; MW designed the study and edited the final version of the manuscript. All authors read and approved the final manuscript.

## CONFLICT OF INTEREST

The authors declare that they have no competing interests.
