# Supplementary figures and images for: Amyloid‐beta 1‐40 is associated with alterations in NG2+ pericyte population ex vivo and in vitro
Source: Aging Cell. 2018 Feb 17;17(3):e12728. doi: 10.1111/acel.12728 (PMC5946076; doi:10.1111/acel.12728)

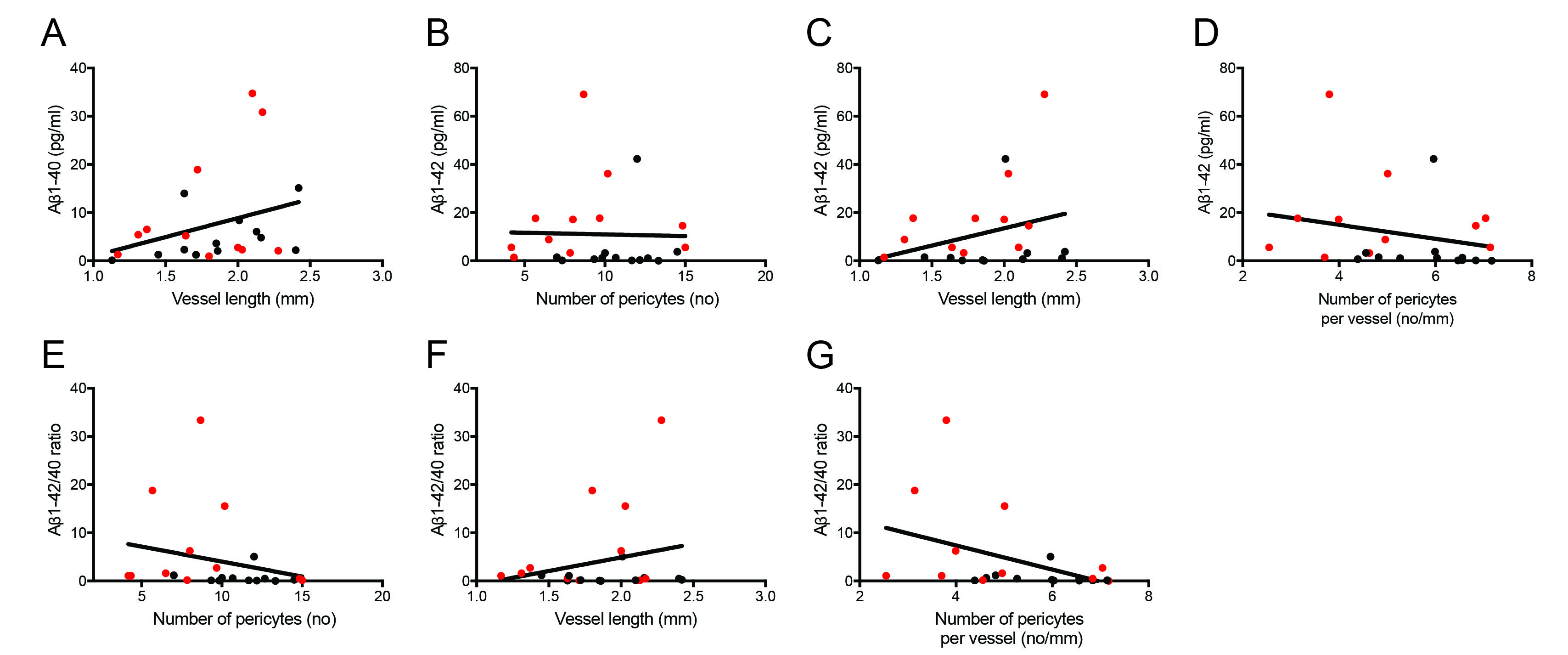

Supplement: Supplementary file 1 [file ACEL-17-e12728-s001.jpg]

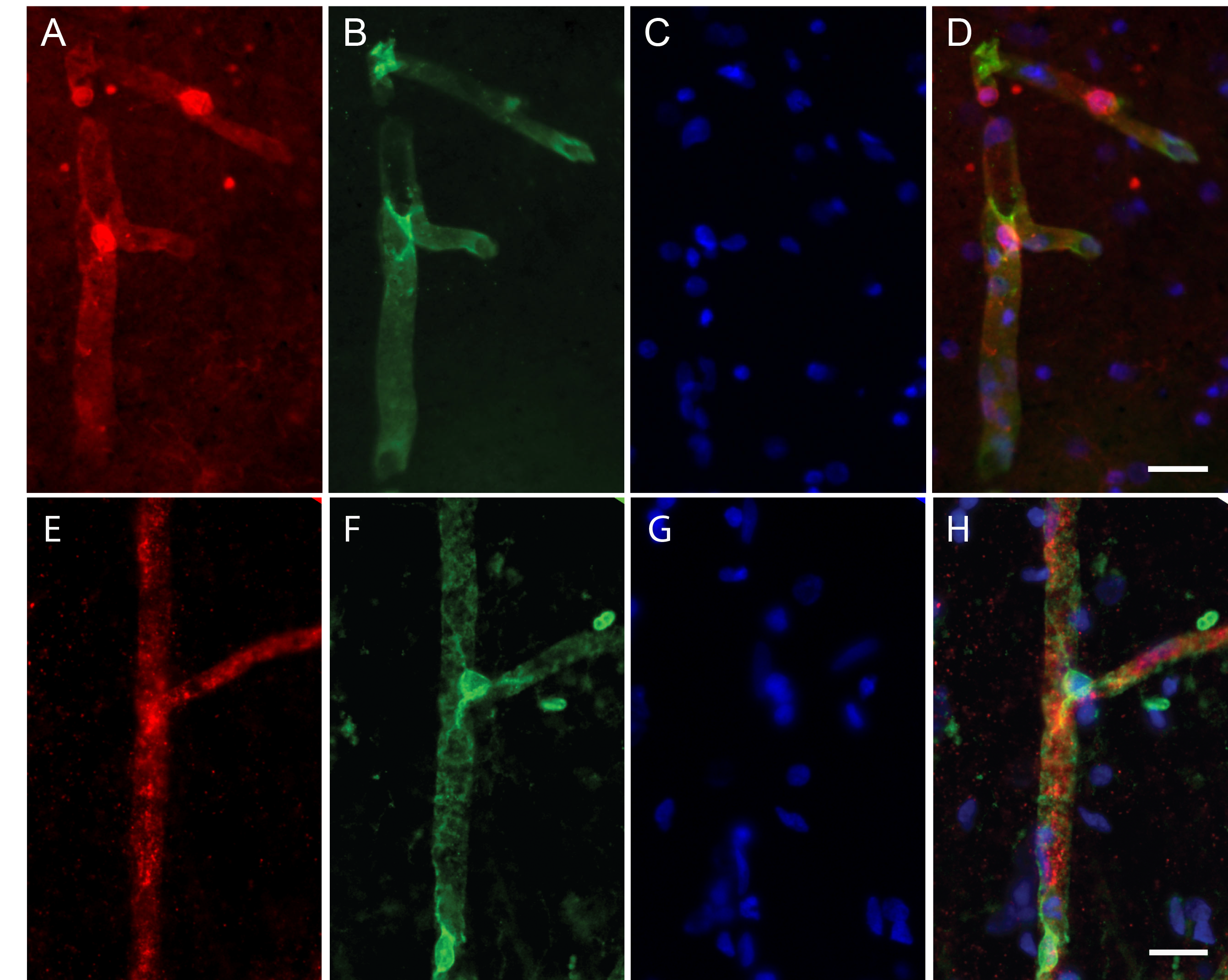

Supplement: Supplementary file 2 [file ACEL-17-e12728-s002.tiff]

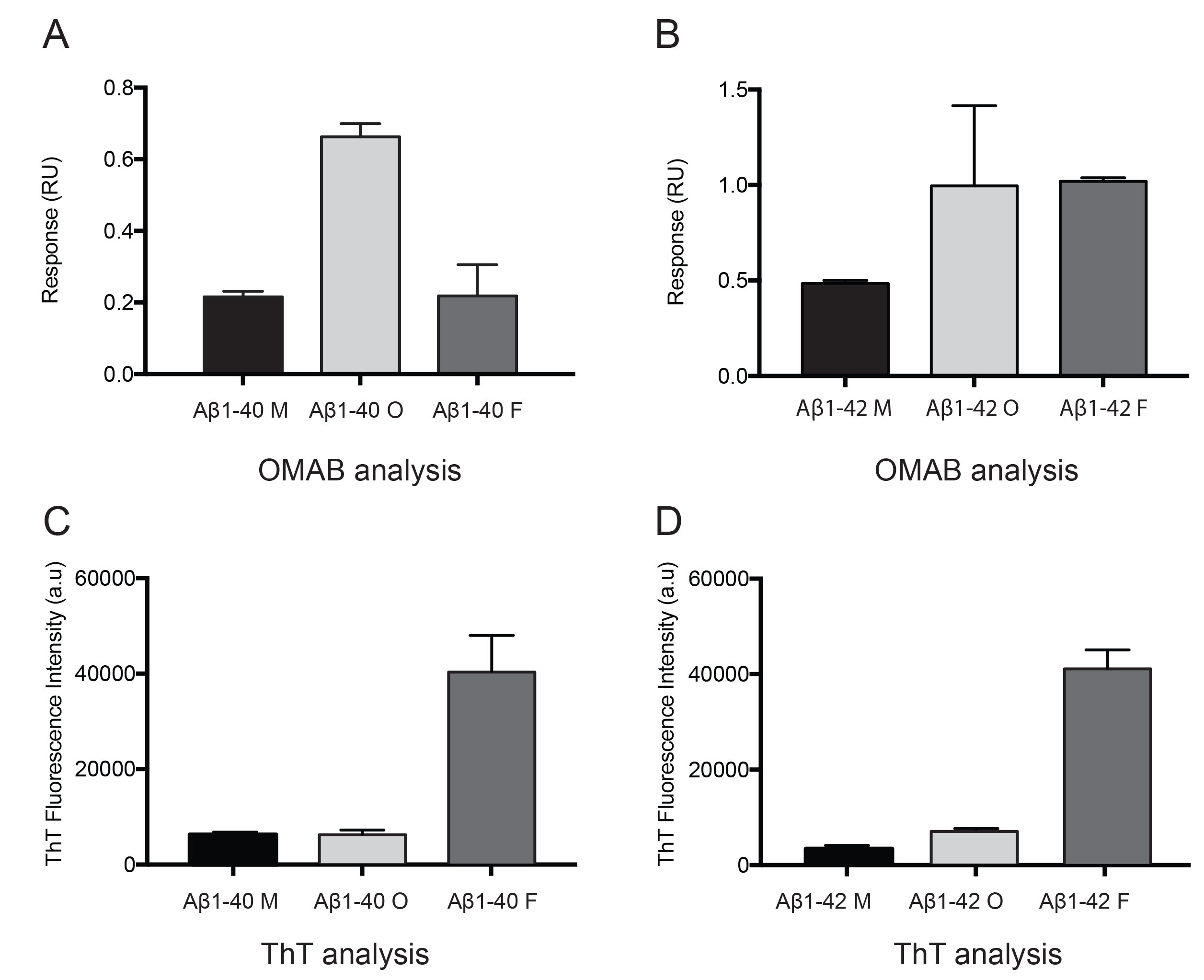

Supplement: Supplementary file 3 [file ACEL-17-e12728-s003.jpg]

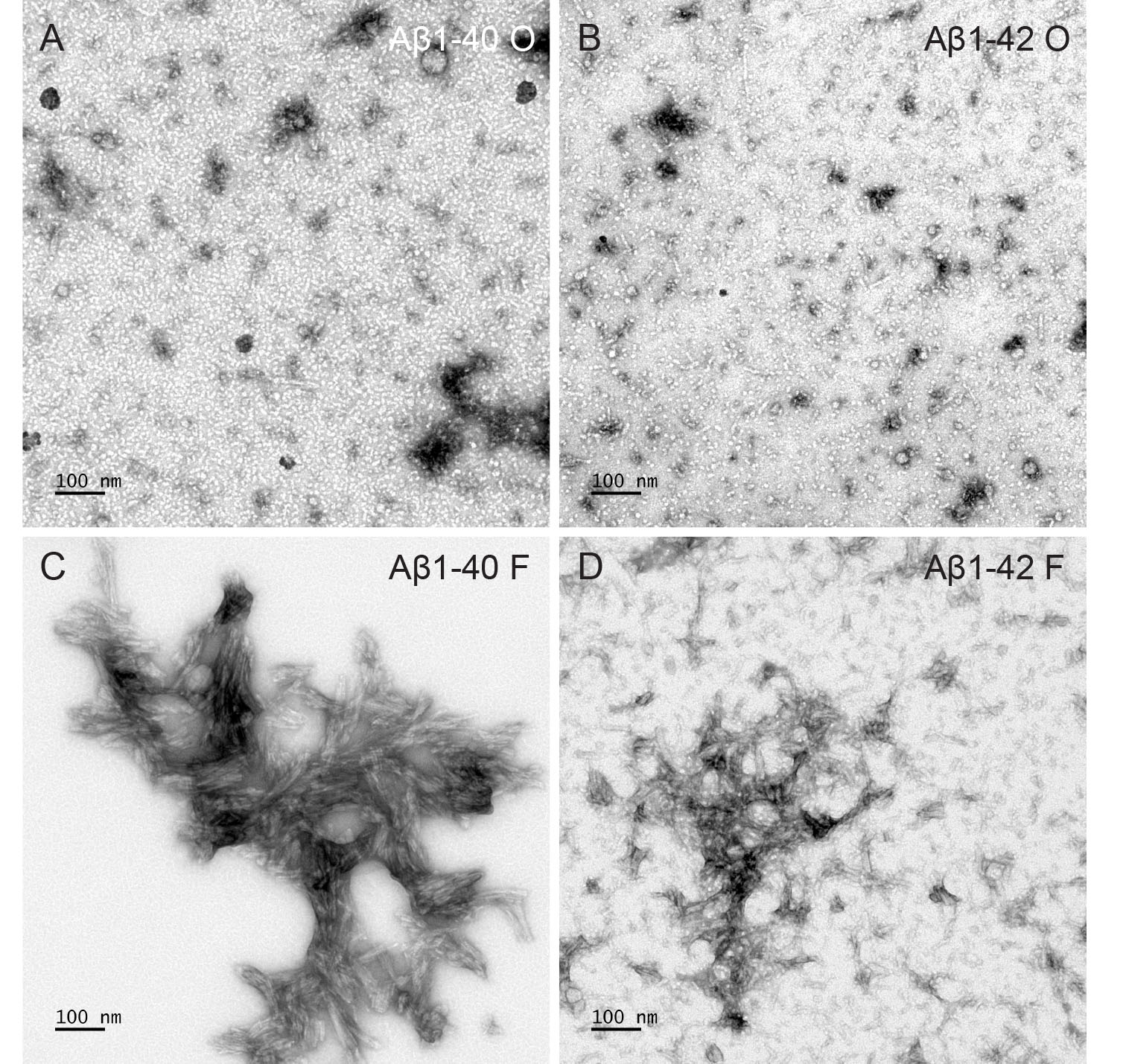

Supplement: Supplementary file 4 [file ACEL-17-e12728-s004.jpg]
